# Sensory representation of an auditory cued tactile stimulus in the posterior parietal cortex of the mouse

**DOI:** 10.1038/s41598-018-25891-x

**Published:** 2018-05-17

**Authors:** Hemanth Mohan, Yasir Gallero-Salas, Stefano Carta, João Sacramento, Balazs Laurenczy, Lazar T. Sumanovski, Christiaan P. J. de Kock, Fritjof Helmchen, Shankar Sachidhanandam

**Affiliations:** 1 Brain Research Institute, University of Zurich, Zurich, Switzerland; 2 Department of Integrative Neurophysiology, Center for Neurogenomics and Cognitive Research, VU University Amsterdam, Amsterdam, The Netherlands; 3 Neuroscience Center Zurich, Zurich, Switzerland; 4 Department of Physiology, University of Bern, Bern, Switzerland; 5 Present Address: Cold Spring Harbor Laboratory, Cold Spring Harbor, NY, USA

## Abstract

Sensory association cortices receive diverse inputs with their role in representing and integrating multi-sensory content remaining unclear. Here we examined the neuronal correlates of an auditory-tactile stimulus sequence in the posterior parietal cortex (PPC) using 2-photon calcium imaging in awake mice. We find that neuronal subpopulations in layer 2/3 of PPC reliably represent texture-touch events, in addition to auditory cues that presage the incoming tactile stimulus. Notably, altering the flow of sensory events through omission of the cued texture touch elicited large responses in a subset of neurons hardly responsive to or even inhibited by the tactile stimuli. Hence, PPC neurons were able to discriminate not only tactile stimulus features (i.e., texture graininess) but also between the presence and omission of the texture stimulus. Whereas some of the neurons responsive to texture omission were driven by looming-like auditory sounds others became recruited only with tactile sensory experience. These findings indicate that layer 2/3 neuronal populations in PPC potentially encode correlates of expectancy in addition to auditory and tactile stimuli.

## Introduction

Sensory processing carried out by the mammalian neocortex allows for the perception of the external environment. While the primary sensory cortices are efficient at processing their respective sensory modality, higher-order sensory association cortices piece together multiple sensory inputs to generate a more comprehensive representation of the outside world. In rodents, the posterior parietal cortex (PPC) is one such association area that lies sandwiched between the primary somatosensory, auditory and visual cortices and has dense reciprocal connections with these regions^1–4^. As such, PPC is capable of processing somatosensory, visual and auditory information, as well as combined stimuli resulting in multisensory enhancement^5–8^. PPC is also part of the attention network implicated in top-down stimulus selection, that is distributed across the fronto-parietal-collicular areas^9^. It is reciprocally connected to the orbitofrontal cortex, anterior cingulate cortex and the frontal eye field (FEF)^1,10^, cortical areas that are involved in attention.

The capacity of PPC to process multimodal stimuli renders it particularly important in coding for peripersonal space, in egocentric and allocentric reference frames^1,11–13^. In addition to playing a role in spatial navigation, PPC is also implicated in sensorimotor decision making in both primates and rodents^14–17^ and in goal-distance estimation with auditory cues^18^. Recently it has also been shown that PPC has a temporal associative role where it encodes the memory of past behavioural choices^19^, as well as choice outcomes and that it can bias action selection based on this history^20,21^. Lesion studies show that damage to the PPC results in failure to attend and respond to contralesional sensory stimuli. This hemispatial neglect occurs despite intact sensory processing at the primary sensory cortices^22^. It can be alleviated partially by the presentation of auditory cues to direct attention to the contralesional side^23^, effectively bringing the unattended sensory stimulus into conscious perception by activating the fronto-parietal network^24^. While visual and auditory processing in PPC during behaviour have been extensively studied in mice^7,15,17,18,25,26^, we know little about how whisker-based tactile stimuli are represented in the neuronal microcircuits of PPC.

Here we exposed naïve awake mice to tactile stimuli in the form of texture touches to the intact whisker pad^27^, presaged by auditory cues. We report that layer 2/3 neurons of PPC show diverse responses to both the tactile stimuli and the auditory cues that precede their delivery. Moreover, we find that omission of the texture stimulus following the auditory cues (in so-called ‘Catch-trials’) elicits responses in a subset of neurons that were largely non-responsive to or suppressed by the texture stimuli. Some of these neurons became responsive to texture omission only after tactile stimulation was coupled with the auditory cues. Others, however, responded to Catch-trials in mice that were exposed to the auditory cues without prior texture-touch experience. In these mice the responses apparently arose from the looming-like auditory sounds and were further enhanced to reporting texture omission upon coupling with texture touch. These findings indicate the presence of potential single-cell neural correlates of expectancy in PPC.

## Results

**Figure 1:**
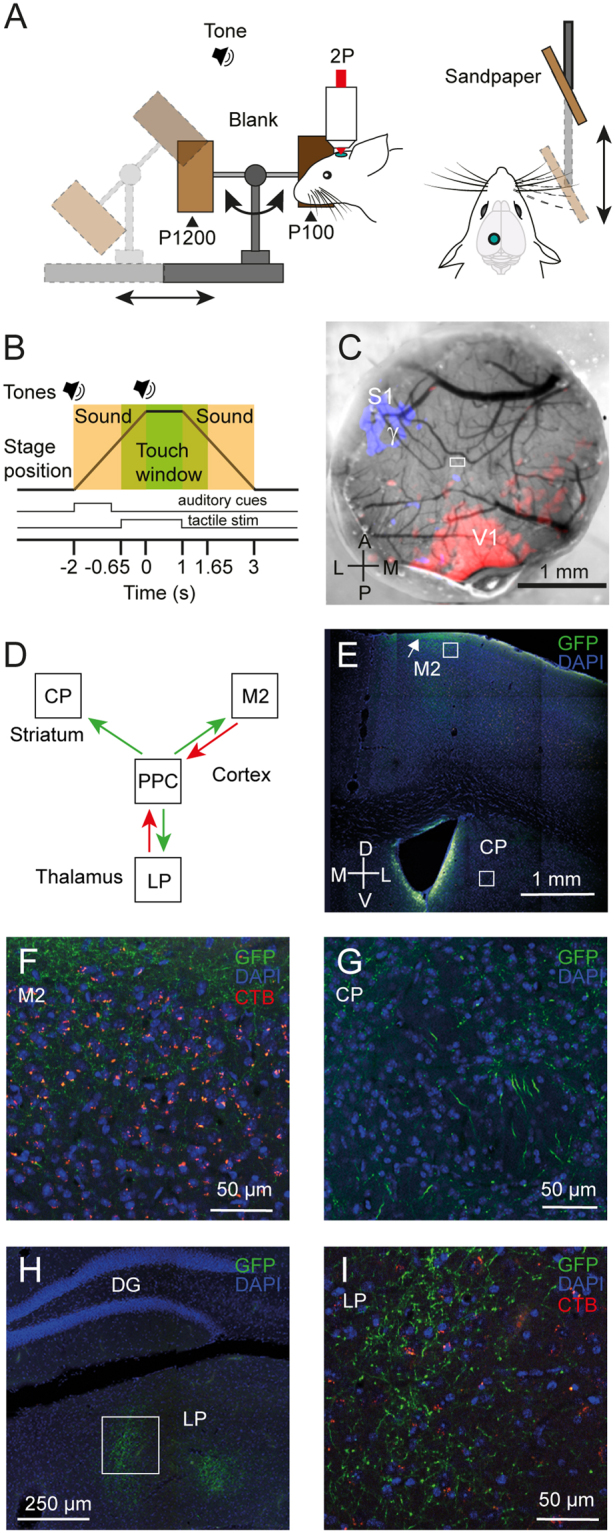
Stimulation setup and localization of PPC in mice. (**A**) Schematic of the texture presentation set-up that allows for 2-photon imaging of the PPC using a cranial window in a head restrained mouse. P100 (rough) and P1200 (smooth) correspond to the graininess of the sandpaper employed. (**B**) Schematic of the trial structure, where the translation stage presenting the textures slides in and out of position. The loudspeaker symbols indicate the 1-ms auditory tones just before the start and at the end of the stage translation, respectively. The shaded beige rectangles indicate the time windows with auditory cues from the translation stage movement and the green rectangle indicates the time window when the textures are in reach for whisker touches. Cue and touch-stimulus vectors used for the analysis windows are shown as well. (**C**) Location of the γ-whisker barrel in S1 and of V1, as determined with IOS (blue and red overlay, respectively) seen through a 4-mm cranial window. The white rectangle indicates the FOV from Fig. 2A. (**D**) Schematic of known anatomical inputs (red arrows) and outputs (green arrows) to PPC. (**E**–**I**) Fluorescence images of retrogradely labelled cell bodies that project to PPC (orange-red) with CTB-Alexa594 (CTB), and anterogradely GFP labelled PPC axonal projections (green), along with DAPI-stained cell nuclei (blue) to determine the anatomical connections of the site identified by IOS. In (**E**) coronal section of the mouse brain displaying M2 and the dorsal striatum (CP, caudate putamen). Note the concentration of the PPC axons in layer 1 of M2 (white arrow). In (**F**) close-up of the region indicated by the white box (upper layer 2/3) at the top of (**E**) with PPC projecting orange-red M2 cell bodies and M2 projecting PPC axons in green. In (**G**) close-up of the region indicated by the white box at the bottom of (**E**) with PPC axons projecting to the dorsal striatum in green. In (**H**) coronal section displaying the dentate gyrus (DG) and LP, with LP-projecting green PPC axons. In (**I**) close-up of the region indicated by the white box in (**H**) with PPC projecting orange-red LP cell bodies intermingled with LP-projecting green PPC axons.

In order to determine how tactile sensory information is represented in the neocortical microcircuit of PPC, we presented one of two textured surfaces (sandpaper of grade P100 and P1200, corresponding to rough and smooth surfaces respectively) to the mouse whisker pad^27^. We adopted a motorized linear stage that randomly presented one of the textures to the whisker pad contralateral to the imaging site (Fig. 1A). We used naïve awake mice pre-implanted with cranial windows and habituated to head restraint in the setup. Before trial initiation and imaging, mice were exposed once to the textures, namely the P100 surface, to set the position of the texture presenter. Two auditory tones signalled the start of the trial and when the texture was in the final position, respectively. Additional auditory cues were generated by the translation motor as it moved in and out of the stimulus position (Fig. 1A, B). To determine the influence of these stage-generated auditory cues, we interleaved the texture trials with ‘Catch’-trials, in which textures were omitted but with all auditory cues present (start and stop tones), including the sounds generated by the stage translation (Fig. 1A; Methods).

In order to localize PPC in mice^3,6^, we performed intrinsic optical signal (IOS) imaging^28,29^ to visualize the boundaries between the γ-barrel column in the mouse whisker barrel cortex and the primary visual cortex V1 (see Methods) (Fig. 1C). This allowed us to select imaging areas in posterior parietal area A^3,30^ in the lateral parietal association cortex (LPtA)^31^, located in between the somatosensory barrel cortex and V1. In four mice, we carried out retrograde and anterograde labelling experiments with tracer injections targeted to LPtA. The resulting labelling patterns validated that this region receives axonal projections from known PPC input sites (Fig. 1D), particularly the thalamic lateral posterior nucleus LP (Fig. 1H,I) and premotor cortex M2 (Fig. 1E-F), as well as sends its axons to known PPC output sites, including M2, the dorsal striatum (Fig. 1E–G) and LP (Fig. 1H,I)^15,32^. Hence, we identify this region between barrel cortex and V1 as part of PPC and subsequently used IOS imaging to guide our selection of imaging areas accordingly.

**Figure 2:**
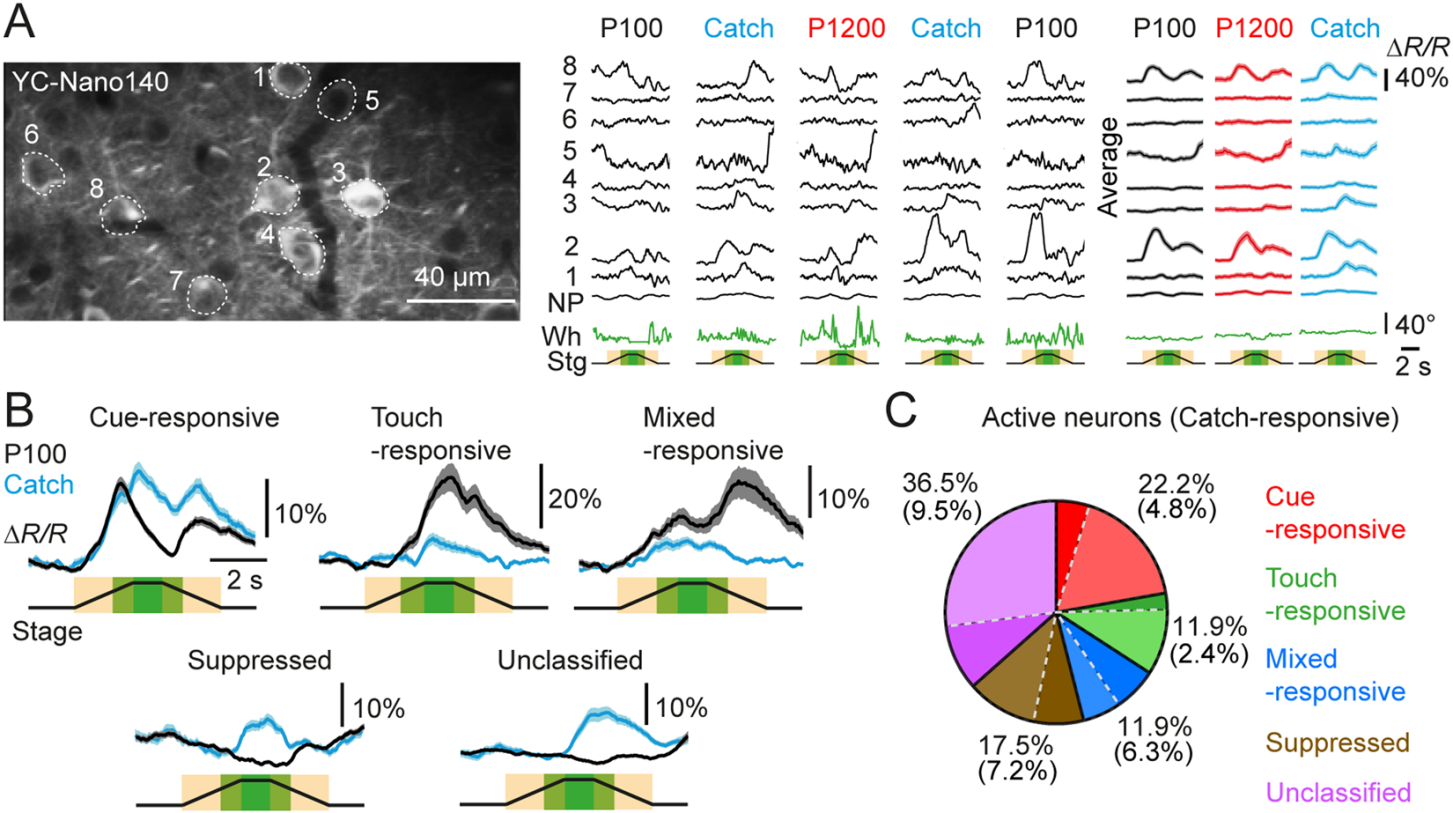
Neural dynamics of sensory representation in the PPC. (**A**) Left, 2-photon image of a FOV with neurons expressing the GECI YC-Nano140. Right, calcium transients from the ROIs selected in the left panel in response to the presented auditory cues and textures, along with the neuropil (NP) signals and the corresponding whisker angles (Wh) in green. The transients represent the very first 5 trials that the mouse has been exposed to. The averaged responses correspond to: texture graininess, P100, n = 57; texture graininess, P1200, n = 26; Catch, n = 17 trials. (**B**) Example average calcium transients of neurons classified as cue-responsive, touch-responsive, mixed-responsive, suppressed and unclassified. Traces are averages of 40 to 60 P100 trials and corresponding Catch-trials. (**C**) Pie Chart illustrating the percentage-wise classification of trial-active neurons and the corresponding distribution of Catch-responsive neurons across the classes (darker slice shade; percentage fractions in brackets). Shaded areas around the mean calcium transients correspond to the standard error of the mean (s.e.m.) here and in all subsequent figures.

We performed *in vivo* calcium imaging of layer 2/3 PPC neurons (at 150 to 220 µm depth) through a cranial window using YC-Nano140, a genetically encoded ratiometric calcium indicator that reliably reports action potential discharge^27^ (Fig. 2A). We acquired imaging data from 13 fields of views (FOVs) in 4 mice, recording calcium signals in a total of 358 neurons. About 35% of neurons (126/358) were active during the trials (see Methods). For each FOV we performed imaging during 100 trials, comprising approximately 40% trials with presentation of each texture and 20% Catch-trials. PPC neurons displayed a diversity of trial-related responses with some neurons responding to the auditory cues, to the texture stimuli, or both, while others showed suppressed activity during cue and texture presentation (Fig. 2A,B). In particular, the auditory-cue-responsive neurons ramped up their activity as the translation stage approached the mouse. This ramping up of activity resembled looming responses in neurons observed in the superior colliculus^33^ and auditory cortex^34^, in response to looming visual and auditory cues, respectively. We confirmed that the auditory cue generated by the stage translation was indeed looming-like by performing sound recordings at the location corresponding to the head of the head-fixed mouse (Supplementary Fig. S1). Using a general linear model (GLM) to fit neuronal calcium traces, we classified the trial-active neurons (n = 126) as cue-responsive (22.2%), touch-responsive (11.9%), mixed-responsive (11.9%) and suppressed (17.5%) based on the dynamics of their calcium responses to the trial period; the remaining 36.5% of ‘unclassified’ active neurons responded neither to auditory cues nor to texture presentation (Fig. 2B,C; see Methods). To our surprise, a subset of these ‘unclassified’ neurons (12 out of 46) as well as a subset of classified neurons (26 out of 80) showed a significant response in the touch-stimulus window during Catch-trials, when the texture was omitted (see for example average traces of neurons 1, 3 and 5, Fig. 2A). We call these special neurons ‘Catch-responsive’ neurons (see Methods), which overall made up 30.2% of the active neurons (Fig. 2C). Interestingly, 55.3% of the Catch-responsive neurons were non-responsive or even suppressed during texture presentation trials, suggesting that they were inhibited by the looming-like auditory cues and/or a subsequent texture touch, but relieved from inhibition when the texture was absent.

**Figure 3:**
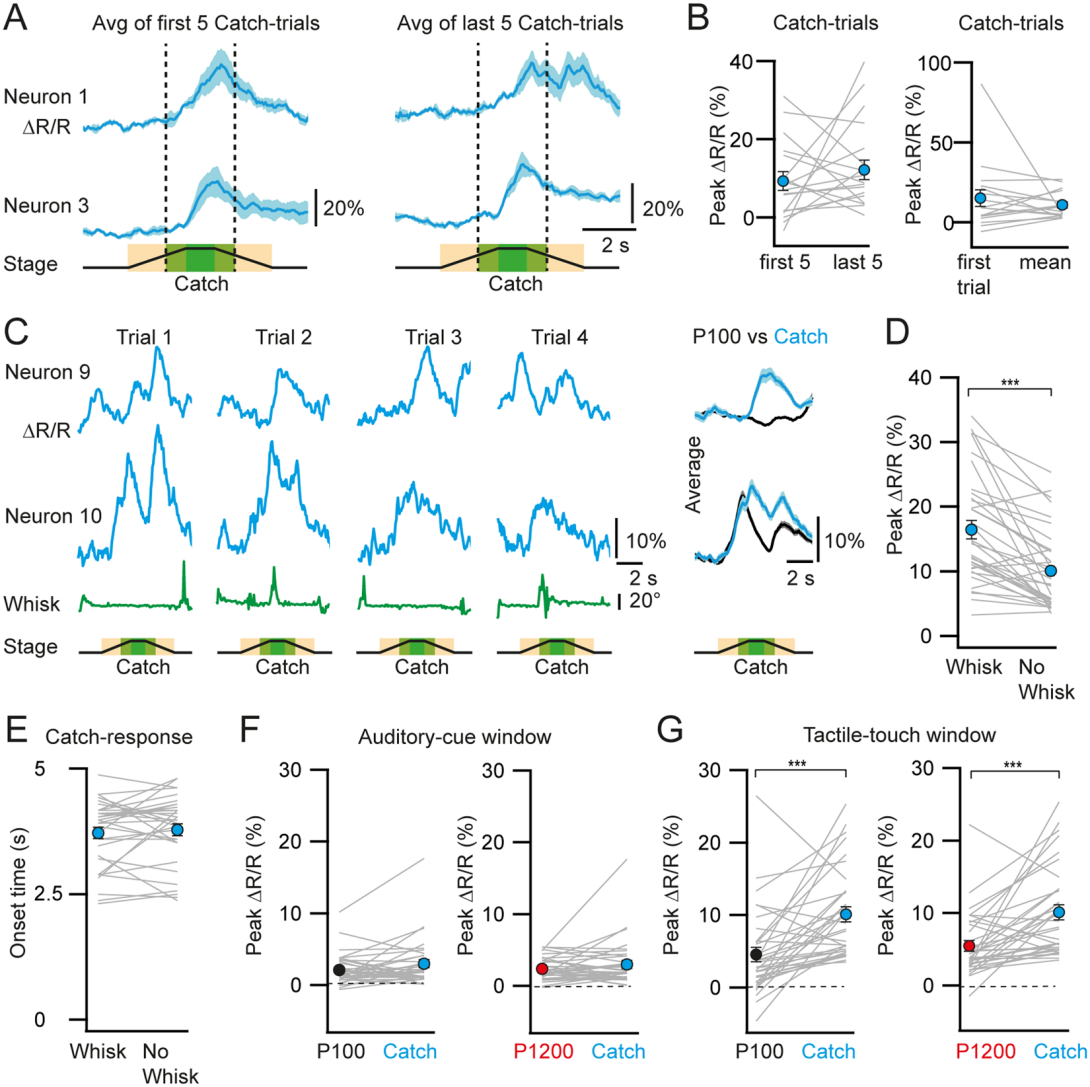
Catch-trial response is robust and stable. (**A**) Example average Catch-trial responses from the first 5 and last 5 Catch-trials. (**B**) Left, mean peak ∆*R/R* of the first 5 and last 5 Catch-trial responses from the first imaging session of each mouse (n = 17 Catch-responsive neurons from 4 mice). Right, mean peak ∆*R/R* of the very first Catch-trial and the mean peak Catch-trial response from all trials of the first imaging session. (**C**) Example single-trial calcium transients of Catch-responsive neurons in trials, with the corresponding whisker angles below, and their average traces (n = 24) compared to their corresponding response to P100 trials (n = 54). Trials 2 and 4 display whisking during the touch-stimulus window. (**D**) Mean peak ∆*R/R* of the Catch-trial response in trials with whisking and no-whisking. (**E**) Mean Catch-response onset times for the same trials in (**D**). (**F** and **G**) Mean peak ∆*R/R* of Catch-responsive neurons to texture trials (P100 and P1200) and Catch-trials in the auditory cue and touch-stimulus windows respectively. Lines represent individual neurons. Filled circles with error bars represent group averages shown as mean ± s.e.m. Statistical significance is indicated by *** for *P* < 0.005. No statistically significant differences were found comparing trials in (**B**,**E** and **F**).

We went on to further characterize these Catch-trial responses by examining their neural dynamics during the course of the experiment. To determine if their activity was experience-dependent and gradually developed over time, we compared the mean peak *ΔR/R* response in the touch-stimulus window between the first 5 and the last 5 Catch-trials for Catch-responsive neurons. The analysis was restricted to the first session, in which a mouse was confronted with the setup (i.e., we only used data from the first imaging area measured in each mouse). We found that the peak amplitudes of the evoked responses in Catch-responsive neurons were comparable at the beginning and end of the imaging session (Fig. 3A,B; peak ∆*R/R*: first 5 trials 9.3 ± 2.4%, last 5 trials 12.1 ± 2.5%; p = 0.75; n = 17 Catch-responsive neurons in 4 FOVs, Wilcoxon signed paired test). Moreover, the peak calcium transient amplitude in response to the very first Catch-trial was comparable to the mean peak response for all Catch-trials (Fig. 3B; peak ∆*R/R*: first trial 15.2 ± 5.2%, trial mean 10.9 ± 1.9%; p = 0.89, n = 17 Catch-responsive neurons in 4 FOVs, Wilcoxon signed paired test). This suggests that PPC neurons are able to report the omission of the texture reliably and stably over time. Furthermore, for all 4 FOVs the first Catch-trial was presented as the second trial in the sequence of trials, suggesting that the PPC is able to rapidly encode the omission of the auditory-cued tactile stimulus.

We next verified whether the Catch-trial responses might reflect an efferent copy of a motor command such as whisking because the PPC has strong reciprocal connections with the motor and premotor cortices^1–3^. Catch-trial responses were observed both in the absence and presence of whisking behaviour (see Methods) during the touch-stimulus window, indicating that they were not solely dependent on whisking (Fig. 3C). A comparison of the peak of the Catch-trial responses in the presence and absence of whisking during this time window revealed that the response was enhanced by whisking (Fig. 3D; peak ∆*R/R*: whisking 16.4 ± 1.4%, non-whisking 10.1 ± 1.0%; p = 1.47 × 10^−7^, n = 34 Catch-responsive neurons in 12 FOVs, Wilcoxon signed paired test; whiskers could not be tracked in one of the 13 FOVs). However, the onset time of the Catch-trial responses (from trial start) were comparable in the presence and absence of whisking (Fig. 3E; Catch-response onset time: whisking 3.7 ± 0.1 s, non-whisking 3.8 ± 0.1 s; p = 0.56, n = 34 Catch-responsive neurons in 17.3 ± 0.1% and 82.7 ± 0.1% of Catch-trials respectively, Wilcoxon signed paired test). Whisking onset and the corresponding Catch-trial onset were also comparable (onset time: whisking 4.1 ± 0.1 s, Catch-response 3.9 ± 0.1 s; p = 0.20, n = 12 FOVs, Wilcoxon signed paired test). While Catch-responsive neurons showed comparable calcium signals during the auditory-cue window, for both Catch-trials and texture presentation trials (Fig. 3F; peak ∆*R/R*: P100 2.1 ± 0.4%, P1200 2.4 ± 0.3%, Catch 3.0 ± 0.6%; p = 0.07 for P100 vs Catch, p = 0.72 for P1200 vs Catch; n = 34 Catch-responsive neurons in 12 FOVs; Wilcoxon signed paired test), Catch trial responses in the touch-stimulus window were larger compared to their corresponding texture-touch responses (Fig. 3G; peak ∆*R/R*: P100 4.5 ± 1.0%, P1200 5.5 ± 0.7%, Catch 10.1 ± 1.0%; p = 1.72 × 10^−5^ for P100 vs Catch, p = 4.66 × 10^−5^ for P1200 vs Catch; n = 34 Catch-responsive neurons in 12 FOVs; Wilcoxon signed paired test). To verify whether Catch-trial responses might have been acoustically driven by the texture stop tone, we also determined the onset times exclusively in Catch-trial responsive neurons that were suppressed or non-responsive to textures, avoiding overlap with auditory-cue or texture-touch responses, and analysed only non-whisking trials. Apart from one neuron (onset 120 ms after the texture stop tone) the others displayed onset times well before the translation stage came to rest (onset 580 ± 65 ms before texture stop tone, n = 18 neurons). This indicates that the Catch-trial responses were not due to the stop auditory tone and could potentially represent an expectancy response that can be further enhanced by whisking.

**Figure 4:**
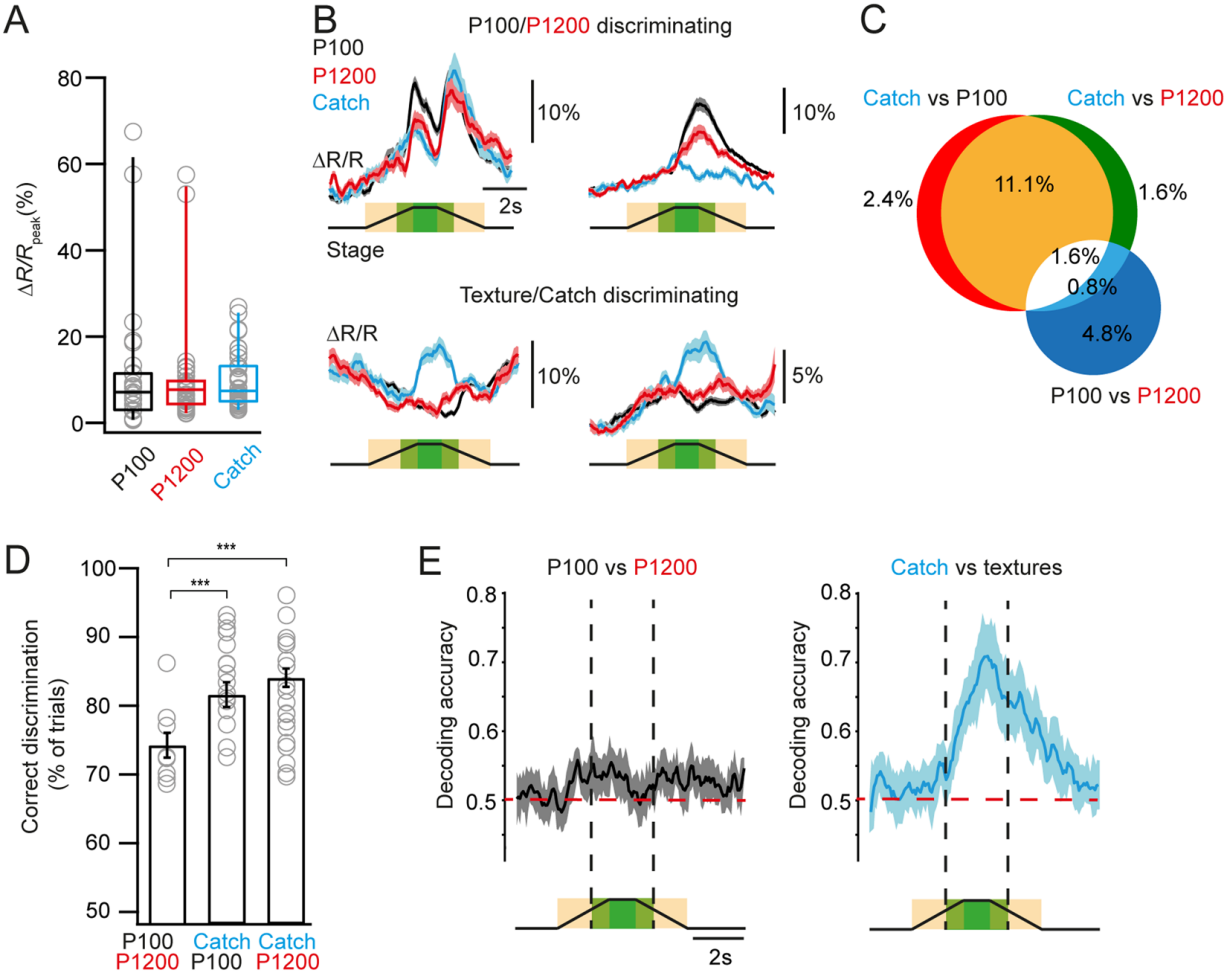
Texture discrimination and omission detection in the PPC. (**A**) Median peak ∆*R/R* of texture evoked responses (touch-responsive and mixed responsive neurons) and Catch-trial responses (Catch-responsive neurons) respectively. (**B**) Example average calcium transients of neurons that can discriminate textures (top) and those that can report texture omission (bottom). Traces are averages of 20 to 50 trials. (**C**) Venn diagram of the distribution of discriminating neurons. (**D**) Performance of neurons discriminating above chance either texture type (left) or presence vs. omission of the texture-stimulus (right). (**E**) Population level decoding accuracy of texture graininess and texture omission. Horizontal dashed red lines represent chance level. Box plots represent the median, the 25^th^ and 75^th^ percentiles in the boxes, whereas the sidebars represent the 5^th^ and 95^th^ percentiles of the distribution. Open circles represent individual neurons. Bar graphs with error bars and traces with shaded areas represent group averages shown as mean ± s.e.m. Statistical significance is indicated by *** for *P* < 0.005. No statistically significant differences were found comparing trials in (**A**).

We then characterized how the two textures and the absence of textures following the auditory cues are represented in PPC. Calcium responses in neurons active during texture presentation trials were comparable to peak calcium activity in Catch-trial responsive neurons during the touch-stimulus window (Fig. 4A; peak ∆*R/R*: P100 11.0 ± 2.7%, P1200 10.3 ± 2.2%, Catch 9.8 ± 1.0%; p = 0.27 for P100 vs Catch and p = 0.13 for P1200 vs Catch, n = 31 touch-responsive and mixed neurons, n = 38 Catch-responsive neurons; Wilcoxon rank test). We carried out an ROC analysis to identify the fraction of neurons that could significantly discriminate between the two presented textures as well as the fraction of neurons that could discriminate between texture-presentation and Catch-trials (compared to shuffled controls, Methods; example traces in Fig. 4B). Among the trial-active PPC neurons 7.2% (9/126) discriminated between the graininess of the presented textures whereas 17.5% (22/126) discriminated between texture presence and omission (Fig. 4C). The subset of Catch-responsive neurons discriminative for presence vs. omission of the texture discriminated a higher percentage of individual trials compared to texture-type discriminating neurons (Fig. 4D; percentage of trials discriminated: P100 vs P1200 74.1 ± 1.9%, Catch vs P100 81.5 ± 1.8%, Catch vs P1200 84.0 ± 1.3%; p = 2.8 × 10^−5^ for P100 vs P1200 and Catch vs P100 and p = 6.5 × 10^−5^ for P100 vs P1200 and Catch vs P1200, n = 9 texture-type discriminating neurons, n = 19 neurons discriminating Catch vs P100, n = 19 neurons discriminating Catch vs P1200; Wilcoxon-Mann-Whitney test; see Fig. 4D for their distribution). As a complement to the ideal observer ROC analysis, we trained a linear support vector machine (SVM) classifier to decode texture graininess and texture omission from single-trial population activity, on a frame by frame basis. This simple readout model could effectively decode texture graininess and texture omission during the touch-stimulus window with about 55% and 70% accuracy, respectively (Fig. 4E). The decoding accuracy was improved when the mean of the touch-stimulus window was used (60.1 ± 3.1% for texture graininess; 73.1 ± 4.3% for texture omission). Overall, these results suggest that texture presence vs omission and to a lesser degree texture graininess are reliably represented in PPC in naïve animals.

**Figure 5:**
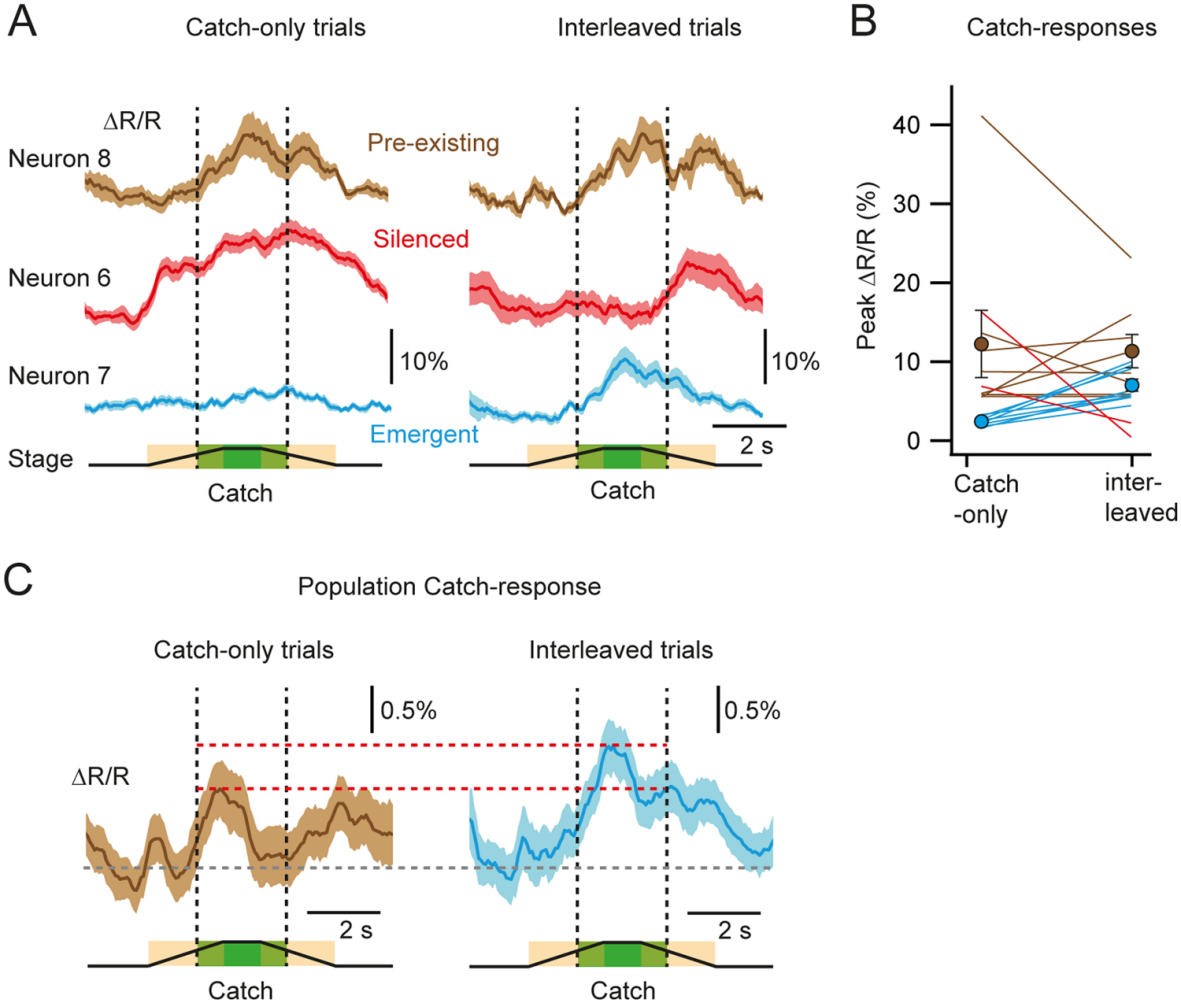
Encoding of auditory-cued texture omission in the PPC. (**A**) Example average Catch-trial responses from pre-existing (brown trace), silenced (red trace) and emergent (blue trace) Catch-responsive neurons from the Catch-only and subsequent texture and interleaved trials. (**B**) Mean peak ∆*R/R* of the Catch-trial response as observed in the touch-stimulus window of pre-existing (n = 8) and emergent (n = 8) Catch-responsive neurons, before (Catch-only) and during (interleaved) tactile sensory experience. The colour codes are identical to (**A**). No mean value is presented for the two suppressed neurons. (**C**) Population Catch-response as observed in the Catch-only trials and after tactile experience in the interleaved trials. Horizontal dashed red and grey lines indicate the mean peak population response in the touch-stimulus window and the baseline, respectively. Lines represent individual neurons. Filled circles with error bars represent group averages shown as mean ± s.e.m.

While the PPC might be capable of rapidly encoding texture omission following tactile sensory experience, our findings do not exclude the possibility that Catch-trial responses are present in PPC neurons prior to the presentation of tactile sensory stimuli. In other words, the Catch-trial response might potentially be generated by the looming-like auditory cue produced by the stage. To verify this possibility, we presented an additional 3 naïve mice first with Catch-trials only, effectively decoupling the auditory cues from tactile stimuli. We imaged layer 2/3 PPC neurons in 2 FOVs per mouse, and imaged 74 trial active neurons. The ‘Catch-only’ trials were followed by the presentation of the two textures interleaved with Catch-trials as in the earlier experiments, where auditory cues were coupled to texture touch (‘Interleaved’ trials). For analysis of this additional data set we excluded trials with obvious whisking behaviour or forepaw and jaw movements, as identified with our behaviour-monitoring camera (see Methods). Indeed, 10 out of the 74 active neurons displayed Catch-responses in the Catch-only trials, as identified using the GLM (see Methods). Eight of these neurons were also Catch-responsive in the interleaved trials (‘pre-existing’ Catch responses) whereas 2 neurons were not classified as Catch-responsive anymore (‘silenced’ responses; Fig. 5A,B). In addition, 8 other neurons were identified as Catch-responsive only after the introduction of tactile stimuli but not in the Catch-only condition (‘emergent’ responses, Fig. 5A,B). Due to the low numbers of Catch-responsive neurons detected, however, more data would be required to determine if these three subsets of neurons represent functionally distinct groups. In order to evaluate the influence of tactile sensory coupling on the Catch-trial response at the single-cell level, we compared the trial-averaged neuronal activity of all neurons in the touch-stimulus window in Catch-only and interleaved trials. The Catch-trial response showed a significant increase (touch-stimulus peak ∆*R/R*: Catch-only trials 3.6 ± 0.7%, interleaved trials 5.2 ± 0.5%; p = 4.8 × 10^−5^, n = 74 neurons, Wilcoxon signed paired test) after tactile experience with 14.9% of active neurons displaying a significant change in activity within this window (8 cells with an increase, 3 cells with a decrease; significance level set at p < 0.01, Wilcoxon-Mann-Whitney test). This was also evident at the population level, where the single-trial population average showed a significant increase in neuronal activity in the touch-stimulus window after tactile experience (Fig. 5C; touch-stimulus peak ∆*R/R*: Catch-only trials 0.95 ± 0.3%, interleaved trials 1.4 ± 0.3%; p = 0.039, n = 74 neurons, Wilcoxon-Mann-Whitney test). In a separate set of experiments in these mice, we were also able to evoke Catch-trial like responses in neurons when presented with the audio playback of the looming-like auditory cue but not when presented with a non-looming recording (Supplementary Fig. S2). Our results suggest that while looming-like auditory cues alone can evoke responses in the PPC, coupling with tactile stimuli enhances this response and further recruits more neurons within PPC that can report subsequent texture-touch omissions.

## Discussion

We found that layer 2/3 neuronal populations in the mouse PPC display a diversity of responses to an induced texture touch, with neurons increasing their activity to the preceding auditory cues and/or tactile stimuli, as well as other neurons being suppressed by these stimuli. Few PPC neurons (7.2%) were able to discriminate the two distinct textures presented. This low discrimination power is not surprising as the experimental sessions were only the second time that the naïve mice were exposed to these textures (the first being setting the texture stop position, prior to imaging start). No explicit association of a relevant context to the texture graininess as stimulus feature or any reinforcement training had been applied. In the somatosensory barrel cortex the proportion of neurons that can discriminate the same texture stimuli as applied here in a reward-based go/no-go task is higher in trained compared to naive animals^27,35^. More interestingly, we found that about 30% of trial-active PPC neurons responded to the omission of the texture touch that always arrived after the auditory cues. This was observed in the very first Catch-trial, suggesting that these neurons either are capable of rapid stimulus binding and association^36^ or that they respond directly to the auditory cues generated by the stage translation. We found evidence for both, as Catch-responsive neurons were observed in both the initial Catch-only trials prior to texture touch as well as emerging later in another set of neurons in the subsequent series of interleaved trials. Coupling tactile stimuli to the auditory cues further enhanced the Catch-trial response to texture omissions. Hence, neurons within PPC are capable of representing a change in sensory flow, in a manner that is both rapid and stable. This could be essential for the role of PPC in spatial processing, navigation and the allocation of attention, allowing it to rapidly update changes in sensory information in particular to objects that are close to the body.

Our stimulation paradigm incorporated an auditory cue that was looming-like, generated by the movement of the translation stage and reported reliably by cue-responsive neurons. Looming stimuli have been shown to be behaviourally salient in humans and primates^37,38^, and more recently in mice as well^34^. Looming sounds have been proposed to represent the approach of the sound source in the context of threat detection, either in the form of an attack or collision, and can evoke rapid attention capture and expectancy^38^. Indeed the multimodal neurons in the ventral intraparietal area (VIP) in primates have been shown to respond strongly to looming stimuli^39,40^. In a more benign context looming sounds can simply indicate the arrival of the source that potentially is associated with a touch event. Hence, in the Catch-only trials (Fig. 5A) as well as in the audio playbacks of the looming-like auditory cue (Supplementary Fig. S2) the Catch-trial responses could potentially represent a neural correlate of expectancy, here for a potential touch event. This information is carried over to the period after sensory experience (by pre-existing Catch-responsive neurons) and its representation further strengthened upon omission of the cued tactile stimulus (by emerging Catch-responsive neurons). It is also plausible that the pre-existing Catch-responsive neurons are purely looming sound responsive in the Catch-only trials and start to report texture omission only after coupling with texture touch. These findings are in line with the recently reported temporal associative role of PPC^19,20^, which could be due to its long timescale population dynamics allowing the combination of events separated in time^26^.

An alternative interpretation is that Catch-responsive neurons are actually acoustically driven and the presence of texture touches merely suppresses their response. Indeed, more than half of Catch-responsive neurons did not respond to or were suppressed by texture touches. However, these Catch-trial responses had onset times well before the tone signalling stage stop (with the exception of one neuron), suggesting that something else than sound, such as the expectancy of an incoming object, evoked the increase in activity. The remaining Catch-responsive neurons that also respond to the cue-tone and touch could represent neurons also sensitive to sound-offset^41^. Our results argue against this notion, however, because sensory experience led to changes in the number of Catch-responsive neurons in subsets of PPC neurons, which should not be the case for a reliable sensory response. Unfortunately, we were unable to remove the looming-like auditory cue, as it is an integral part of the texture presentation apparatus. We were also able to evoke Catch-trial like responses in neurons when presented with a playback of this auditory looming-like cue, and not with a non-looming recording in mice with previous tactile experience, effectively eliminating other potential unaccounted stimulus sources for the evoked response.

Catch-responsive neurons displayed responses that were comparable in amplitude to texture-evoked responses. Furthermore, the omission of texture touch could be reliably decoded at the population level. Hence these neurons could function as reporters of mismatch signals, which could reliably convey information about unexpected changes in sensory flow to the prefrontal cortex, FEF and LP (the mouse equivalent of the pulvinar), brain areas that are implicated in the allocation of attentional resources^9^. Indeed stimulus-omission responses have also been described in the auditory^42,43^ and visual systems^44–46^ in the context of mismatch negativity (MMN) and oddball responses. However, the stimulus presentation paradigms employed in MMN are different compared to those used in our study. More recently, mismatch responses have been reported in mouse V1^47,48^ when visual flow is not correlated with self-initiated locomotion. These mismatch signals were also present in LP inputs to layer 1 of V1^49^, and mediated by a balance of motor-related excitation and visually driven inhibition during coupled sensorimotor experience^50,51^. While our stimulus paradigm does not involve a conflict with a self-initiated stimulus response, such as visual feedback from locomotion, it nonetheless incorporates a behaviourally salient stimulus (the looming auditory cue) that when coupled with tactile stimuli, can lead to a rapid encoding of omissions in the coupled sensory flow. LP, along with the posterior nucleus Po, is the main source of thalamic input to the PPC, which does not receive any direct input from primary sensory thalamus^2,52^. Hence, LP/Po could be a potential source for the Catch-trial responses that we have observed in layer 2/3 of the PPC. This could be in turn modulated through the activation of local inhibitory circuits by top-down feedback, potentially from the FEF, providing the prediction signal in the proposed framework of predictive coding^53^.

## Methods

### Viral and tracer injections

Experimental procedures followed the guidelines of the Veterinary Office of Switzerland and were approved by the Cantonal Veterinary Office in Zurich. Intrinsic optical signal (IOS) imaging was performed on young adult (P35-42) male wild-type C57/BL6 mice under light isofluorane anaesthesia to identify the location of PPC by exclusion. In brief, to avoid the activation of surrounding whiskers, all whiskers except the γ-barrel-column whisker on the right whisker pad of the mice were trimmed and their locations were mapped using IOS on the exposed skull under 625 nm illumination during whisker stimulation (rostrocaudal deflections at 10 Hz). The location of primary visual cortex (V1) was similarly mapped using full-field stimulation with a green LED placed 5 mm in front of the contralateral eye. Subsequent viral or tracer injections were targeted to the non-activated region between these two identified sites, within 500-μm from the border of the IOS corresponding to the γ-barrel-column location (light blue overlay in Fig. 1C). To verify that we are able to localize PPC by IOS exclusion as described above, we went on to verify the established anatomical input and output connections to PPC (n = 4 mice). To identify the sites that send their axons to PPC, the retrograde tracer CTB-Alexa594 (Molecular Probes, Invitrogen; 200 nl, 1% wt/vol) was injected into PPC ~300 μm and 500 μm below the pial surface targeting layer 2/3 and layer 5, respectively. A similar injection with AAV8-hSyn-Jaws-GFP-ER2 (200 nl, UPenn) was performed in PPC to follow anterogradely labelled axons and identify output regions that PPC projects to. Note that both injections were performed at the same injection site but spaced by at least 2 weeks (AAV8 followed by CTB), to allow for sufficient expression and uptake respectively. For calcium imaging, AAV1-*EF1α-YC-Nano14*0 (300 nl, ~1 × 10^9^ vg/μl) was injected into the PPC (n = 7 mice), targeting layer 2/3 (~300 μm below the pial surface) to induce expression of the ratio-metric genetically encoded calcium indicator yellow cameleon Nano-140 (YC-Nano140)^54^.

### Verification of PPC location

Following 5–7 days after CTB-Alexa594 injection to allow for uptake^55^, and 3 weeks in the case for virus expression of GFP, mice were anesthetized (ketamine/xylazine; 100/20 mg/kg body weight) and perfused transcardially with 4% paraformaldehyde in phosphate buffer, pH 7.4. Coronal cortical sections (50-µm thickness) were cut using a vibratome (VT100; Leica). Slices were stained with 2.5 μM DAPI for 10 min and mounted on glass slides. Images were acquired with a confocal microscope (Fluoview 1000, 20× and 10× objectives; Olympus) with green (GFP), red (CTB-Alexa594), and blue (DAPI) excitation/emission filters.

### Cranial window implantation and habituation

In order to carry out long-term *in vivo* calcium imaging, a cranial window was implanted 24 hrs after virus injections over PPC as described previously^56^. Briefly, a craniotomy was performed at the injection site. A cover glass (4 mm diameter) was placed directly over the exposed dura mater and sealed to the skull with dental acrylic. A metal post was fixed to the skull with dental acrylic, contralateral to the cranial window, to allow for subsequent head fixation. One week following chronic window implantation, mice were handled daily for 1 week, and gradually habituated to head fixation. This also gave time for the trimmed whiskers to re-grow. Mice were subsequently used for texture presentation only if their whisker pads were fully intact.

### Texture presentation

Behaviour experiments were performed using a data acquisition interface (USB-6008; National Instruments) and custom-written LabVIEW software (National Instruments) to control devices required for texture presentation. Commercial-grade sandpaper (3 M) was used, with a rough sandpaper (P100) and smooth sandpaper (P1200) as stimuli. Sandpapers were mounted onto panels attached to a stepper motor (T-NM17A04; Zaber) mounted onto a motorized linear stage (T-LSM100A; Zaber) to move textures in and out of reach of whiskers of head-restrained mice. The textures were presented in a randomized order, with no more than 3 consecutive presentations of the same texture. Trials without texture presentation (“Catch”) but with linear stage translation were interleaved (Fig. 1A). In a separate set of experiments, Catch-trials were presented first, followed by interleaved texture and Catch-trials (Fig. 5). A trial consisted of a 2-s pre-stimulus period, a 2-s translation period of the linear stage to bring the texture in contact with the whiskers where it remained for 1-s before retraction. Two 2093-Hz auditory cue tones (1 ms) signalled the start and stop of the stage translation, respectively (Fig. 1B). Stage translation itself generated a sound, and served as an additional auditory looming cue that indicated the arrival of the incoming texture. No sounds were presented during the 1-s when the textures were stationary, in contact with the whiskers, and correspondingly in the Catch-trials as well. All experiments were conducted in the dark, apart from illumination of the whisker field with an infrared LED (see below). Mice were not required to perform any task during the trials and were not rewarded during texture presentation.

### Sound recordings and presentation

Sound recordings were performed with a microphone (Audio Teck PRO42) placed in the position where the mouse’s head would have been, as well as next to the fixed stage motor that remained stationary while the stage translated, to record the auditory cues generated by stage translation, and amplified with an audio amplifier (SM Pro Audio Q Pre). Note that the 1 ms auditory cue tunes (see above) were not played and recorded here. These audio recordings (looming and non-looming respectively) were then played back via loud speakers from a PC, at 55 dB for both recordings, in the absence of the auditory cue tones.

### Two-photon calcium imaging

We used a custom-built two-photon microscope controlled by HelioScan^57^, equipped with a Ti:sapphire laser system (~100-fs laser pulses; Mai Tai HP; Newport Spectra Physics), a water-immersion objective (16 × LWDPF, 0.8 NA; Nikon), galvanometric scan mirrors (model 6210; Cambridge Technology), and a Pockel’s Cell (Conoptics) for laser intensity modulation. For calcium imaging, we chose the ratio-metric GECI YC-Nano140 as it allows for the correction of movement artefacts in the Z plane typically observed in awake recordings, which can otherwise give rise to the detection of false positive active cells from out of focus planes. Furthermore, YC-Nano140 has a near linear relationship between the amplitude of the calcium transient and the number of action potentials, as verified by single-cell electrophysiology^27^, unlike the GCaMPs. YC-Nano140 was excited at 840 nm and fluorescence collected with blue (480/60 nm) and yellow (542/50 nm) emission filters for CFP and YFP fluorescence detection, respectively. Images were acquired at 15.6 Hz with 128 × 64-pixel resolution. This gave rise to a relatively small field of view, but acquired with a high frame rate to enhance the reliability of detecting neuronal firing. Single trials of 9 to 10-s duration were recorded at a time with 1-s breaks in between trials to allow for hard disk storage during inter-trial interval periods.

### Whisker tracking and behaviour monitoring

The whisker field was illuminated with 940-nm infrared LED light and movies were acquired at 200 Hz (500 × 500 pixels) using a high-speed CMOS camera (A504k; Basler). The time course of the whisker angle across all imaged whiskers was measured using automated whisker tracking software^58^. Whisking amplitude, defined as the angle between the whisker shaft and sagittal plane from nose tip to the back of the head, was used as a measure to represent both rhythmic and non-rhythmic forms of whisking behaviour. We used custom written MATLAB (Mathworks) scripts to extract whisking episodes by first applying a fourier transform on traced theta to identify whisking events with frequences between 4 Hz and 20 Hz. Events with power greater than a predefined threshold were used to segregate significant whisking episodes. Time periods from these whisking episodes was used to define trial events with or without whisking behaviour during the stimulus window. For all trials, the first and last time point for whisker-to-texture contact was quantified manually through visual inspection. In Fig. 5, the presented data comes from trials free of whisking behaviour, forepaw and jaw movements, as identified visually from the movies that had large field of views (FOVs) covering the entire head and frontal torso of the mouse.

### Calcium imaging analysis

The CFP and YFP fluorescence channel data were imported into MATLAB for processing. First, background was subtracted on each channel (bottom 1^st^ percentile fluorescence signal across entire video). Motion correction to both channels was performed by using image registration with TurboReg^59^. Regions of interests (ROIs) corresponding to individual neurons were manually selected from the average intensity projections of a single-trial time series using ImageJ (National Institute of Health) and custom scripts in MATLAB. Mean pixel value for each ROI was extracted for both channels. Calcium signals were expressed as relative YFP/CFP ratio change *ΔR/R* = (*R* − *R*_0_)/*R*_0_. *R*_*0*_ was calculated for each trial as the bottom 12^th^ percentile of the ratio for the trial. Active neurons were identified by two-way ANOVA with repeated measures of the neuronal calcium signal against the neuropil signal, defined by all pixels not assigned to neuronal soma of the overall ROI annotation (significance value, *P* < 0.01). Additionally, they required mean peak responses larger than 4.5%, corresponding approximately to 2 action potentials^27^. We present peak ΔR*/R* within certain time windows as an approximate measure of neuronal activity during this period.

### GLM-based neuronal response classification

A general linear model (GLM) was used to fit the neuronal calcium transients and classify neurons based on their responses^60,61^. The model consisted of four regressors corresponding to the auditory cue (*X*^*ac*^), texture-stimulation (*X*^*ts*^), texture omission in Catch-trials (*X*^*ch*^), and the post-stimulus phase during texture retraction (*X*^*ps*^), respectively, along with their corresponding regression coefficients ${\beta }^{x}$. We did not distinguish the two types of textures to fit the GLM. Each regressor was represented as a vector with the same length as the calcium traces, containing a value of 1 in the frames spanning the respective temporal window and zero-entries otherwise. The texture regressor was zero for the Catch trials and, vice versa, the Catch regressor was zero for texture-stimulation trials. To take into account temporal filtering caused by calcium indicator kinetics, each regressor was convolved with a kernel *H*(*k*) describing the single-action potential evoked calcium transient of YC-Nano140 as a function of frame number *k* with signal amplitude *A* (4.5% ΔR/R), frame rate *fr* (15.6 Hz), onset time constant τ_*on*_ (0.19 s), and decay constant τ_*decay*_ (0.68 s) as parameters^27^:

1 $$H(k)=A(1-{e}^{-(k-1)/(fr\cdot {\tau }_{on})}){e}^{-(k-1)/(fr\cdot {\tau }_{decay})}$$

For each cell, *ΔR/R* traces were concatenated and then fit with the *fitglm* function in MATLAB using the following equation:

2 $$\Delta R/R={\beta }^{0}+{\beta }^{ac}{X}^{ac}+{\beta }^{ts}{X}^{ts}+{\beta }^{ch}{X}^{ch}+{\beta }^{ps}{X}^{ps}$$

A regression coefficient was considered significantly deviating from baseline ${\beta }^{0}$ when the corresponding p-value was below a significance level of α = 0.01. Neurons were considered ‘cue-responsive’ if ${\beta }^{ac}$ and ${\beta }^{ps}$ were both significantly larger than baseline. Similarly, neurons were classified as ‘texture-responsive’ when ${\beta }^{ts}$ was significantly increased. Furthermore, neurons with both ${\beta }^{ac}$ and ${\beta }^{ts}$ significantly greater than ${\beta }^{0}$ were classified as ‘mixed-responsive’. Neurons with regression coefficients ${\beta }^{ac}$ and ${\beta }^{ts}$ significant and smaller than ${\beta }^{0}$ were classified as ‘suppressed’ neurons. The remaining neurons were classed as ‘unclassified’. Of all these neurons, those with ${\beta }^{ch}$ significant and greater than ${\beta }^{0}$ were additionally classified as ‘Catch-responsive’. In order to avoid erroneously classifying cue-responsive neurons with large transients as Catch-responsive, neurons were excluded from being considered ‘Catch-responsive’ if the average calcium transient fell by more than 50% of the peak response within the cue window once the stage stopped moving and failed to show a rise during the touch-stimulus window. Based on this classification scheme, active neurons were placed into 1 of 5 classes (cue-responsive, touch-responsive, mixed-responsive, suppressed and unclassified) and could be additionally classified as Catch-responsive neurons. We obtained an average *R*^2^ value of 0.10 ± 0.01 for all the classified neurons. The GLM was fitted with 100 trials (approximately 80 texture trials and 20 Catch-trials) per neuron for Fig. 2 and approximately 20 Catch-only and 100 interleaved trials (approximately 80 texture trials and 20 Catch-trials) in Fig. 5. With α set to 0.01 the GLM potentially misclassifies 1 neuron each, out of the 126 and 74 trial-active neurons in Figs 2 and 5 respectively. In the case of Catch-responsive neurons, this would correspond to a false positive probability of 3.3% (1 in 38 neurons, Fig. 2), and 10% to 6.3% (1 in 10 and 1 in 16 Catch-responsive neurons before and after sensory coupling, Fig. 5). Similarly, for the classification of cell response types, this would correspond to a false positive probability of 6.7% to 2.2% (1 out of 15 for touch/mixed responsive neurons and 1 out of 46 for unclassified neurons).

### Single-neuron discriminability analysis

To quantify single-neuron discrimination performance we resorted to a receiver operating characteristic (ROC) analysis. This allowed us to quantify the performance achievable by an ideal observer with access only to the mean calcium transients registered during the time window of interest (e.g., the touch-stimulus period). Briefly, for a given binary discrimination task (e.g., texture P100 vs. P1200), an ROC curve was generated by varying a decision threshold over the entire range of possible values. Discrimination performance was then summarized as the obtained area under the curve (AUC). Statistical significance was assessed by a Monte Carlo permutation test (*N* = 10000 random permutations), where true trial type labels were shuffled. Neurons whose AUC values fell above the 99.5^th^ percentile or below the 0.05^th^ percentile of the shuffled distribution were deemed as discriminating above chance (p < 0.01).

### Trial-by-trial population classifiers

We trained linear support vector machine (SVM) classifiers to decode texture graininess (P100 vs. P1200) and texture omission from single trial population activity. A linear SVM computes a simple thresholded weighted sum of inputs, an operation that is plausibly implementable by actual biological neurons; high linear classifier performance has been suggested as an indicator that the neural representation is such that it can be easily readout by downstream neurons^62^. Independent classifiers were trained for each time bin on the calcium transients of every neuron recorded on a given session (approximately 25 neurons). To avoid overfitting, model parameters and were determined on a training set, consisting of 50% of the recorded trials. Classification performance was assessed by measuring the accuracy on the remaining 50% of the trials that resulted from the initial split of the dataset and were held aside during training. To deal with class imbalance (e.g., when learning to discriminate texture omission trials, which are less numerous) we randomly excluded trials from the class with the largest number of examples. Performance figures were averaged across all FOVs and mice and reported as mean ± s.e.m. This approach provides a lower bound on the actual information carried by the population, and does not preclude that more advanced methods cannot extract additional information. Performance did not qualitatively improve when using a more complex radial-basis function kernel (data not shown). We used a reference SVM implementation provided by the scikit-learn (version 0.18.1) python package^63^.

### Statistical analysis

All data are presented as mean ± s.e.m. unless stated otherwise. Non-parametric tests were performed for all analyses, and the Wilcoxon signed-rank paired test and the Wilcoxon-Mann-Whitney test for paired and unpaired group comparisons respectively and the Wilcoxon signed-rank test for single distributions.

### Data Availability

All relevant datasets are available from the corresponding author upon reasonable request.

## Electronic supplementary material

Supplementary Information
