# Supplementary material for: Sensory representation of an auditory cued tactile stimulus in the posterior parietal cortex of the mouse
Source: Sci Rep. 2018 May 17;8:7739. doi: 10.1038/s41598-018-25891-x (PMC5958066; doi:10.1038/s41598-018-25891-x)
Supplement: Supplementary file 1 — Supplementary Information [file 41598_2018_25891_MOESM1_ESM.docx]

**Supplementary Information**

**Sensory representation of an auditory cued tactile stimulus in the posterior parietal cortex of the mouse**

Hemanth Mohan, Yasir Gallero-Salas, Stefano Carta, João Sacramento, Balazs Laurenczy, Lazar Sumanovski, Christiaan P.J. de Kock, Fritjof Helmchen and Shankar Sachidhanandam.

Supplementary information consists of:

Supplementary Figure S1

Supplementary Figure S2


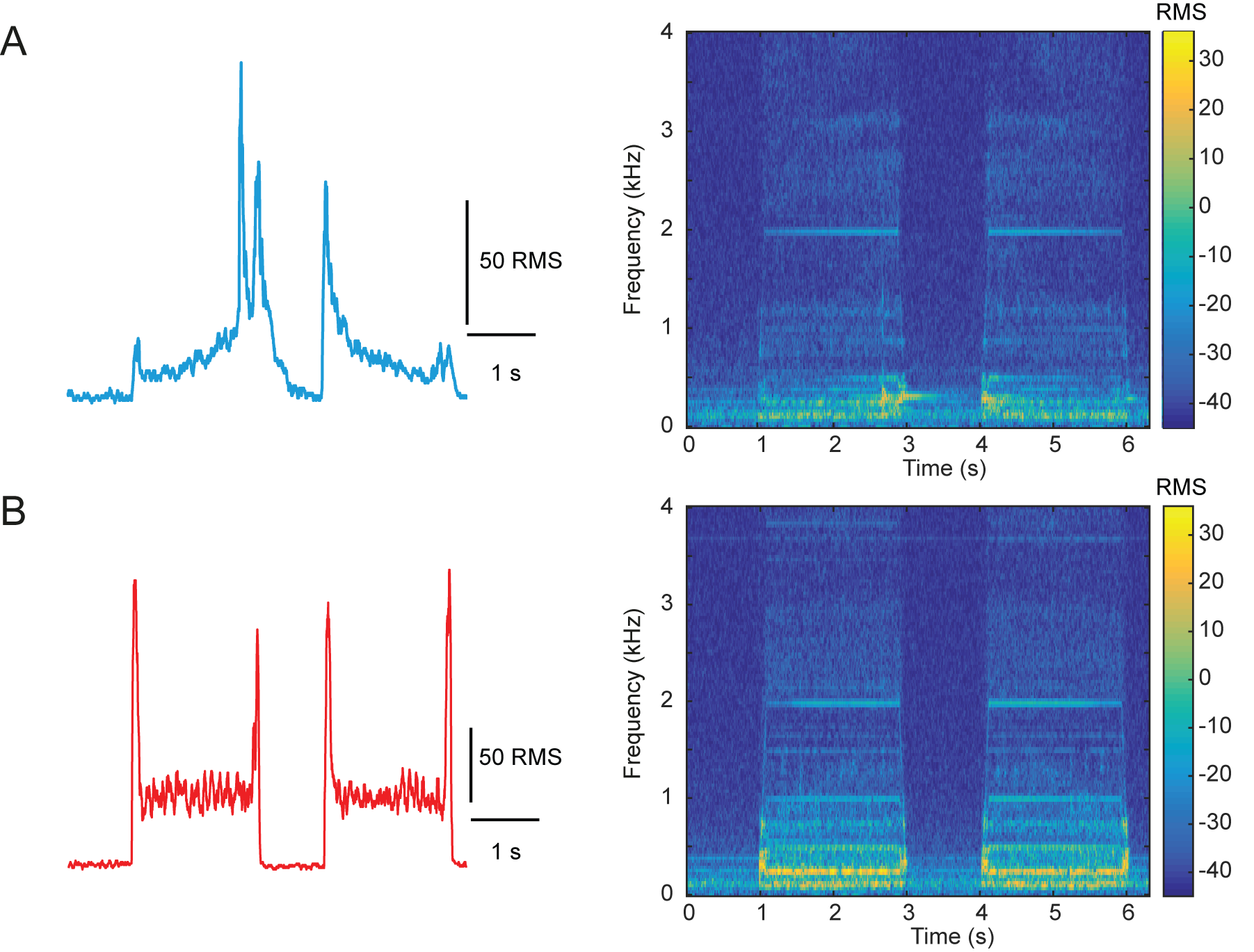


**Supplementary Figure S1.** Looming nature of the auditory cue. ***A*** and ***B***, Sound recordings of the auditory cues (generated by stage translation) and their corresponding spectrograms recorded at the position of the mouse (looming, blue) and next to the stage motor that remains stationary while the stage translates (non-looming, red) respectively. Sound intensity is measured in RMS (root mean square) units.


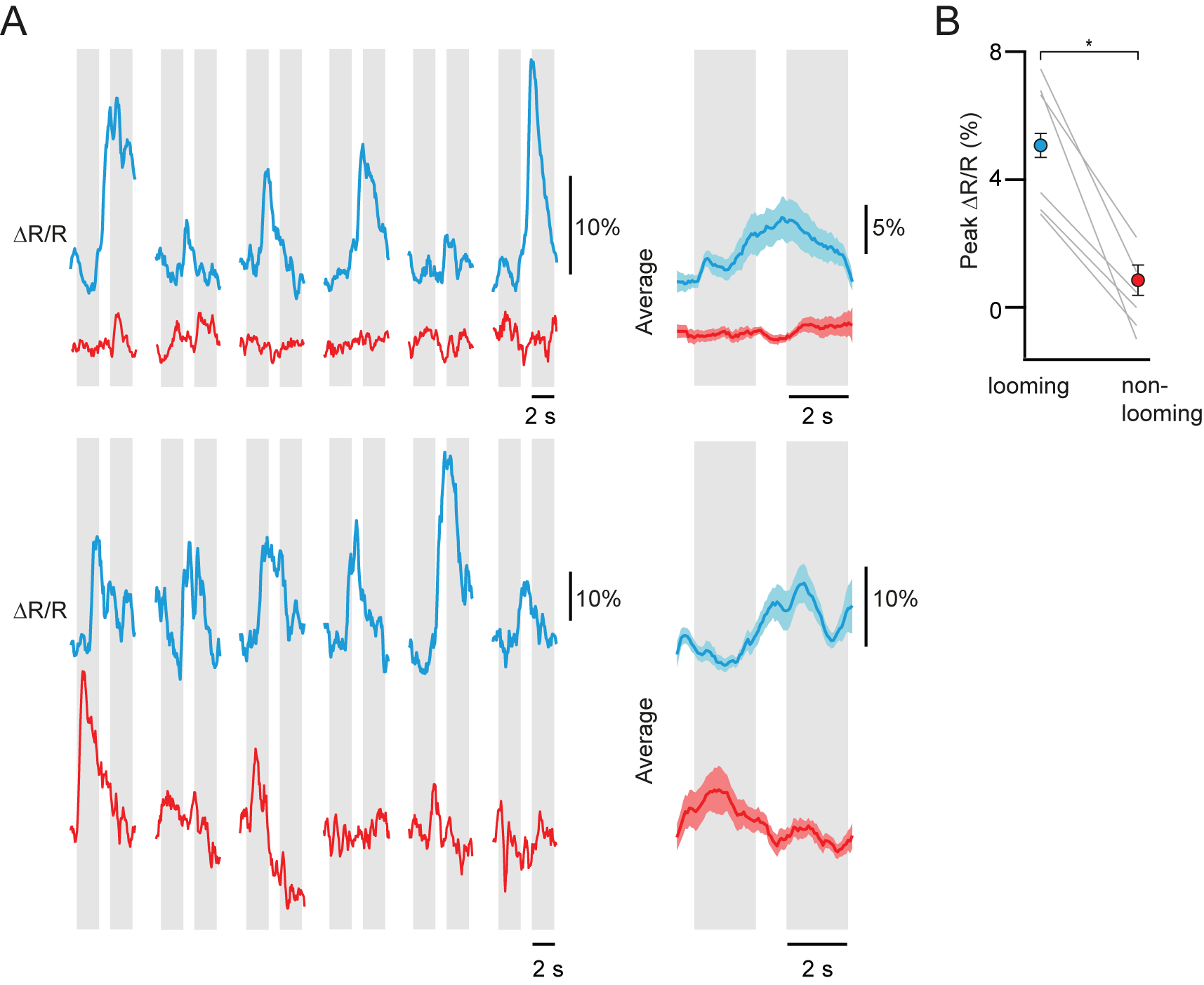


**Supplementary Figure S2.** Catch-trial response can be elicited by looming sounds. ***A***, Single trial neuronal responses from 2 example neurons to the looming (blue) and non-looming (red) audio playbacks, and the corresponding average response. Grey bars correspond to the equivalent stage translation periods. ***B***, Mean peak ∆*R/R* of the audio playbacks (peak ∆*R/R*: looming 5.1 ± 0.9%, non-looming 0.4 ± 0.5%; p = 0.03, n = 6 audio playback responsive neurons in 4 FOVs, Wilcoxon signed paired test). Note that the FOVs are not identical to that in Fig. 5 as we could not locate the same imaged neurons.
